# Nitrate-rich beet juice intake on cardiovascular performance in response to exercise in postmenopausal women with arterial hypertension: study protocol for a randomized controlled trial

**DOI:** 10.1186/s13063-023-07117-2

**Published:** 2023-02-07

**Authors:** Cicero Jonas R. Benjamim, Yaritza Brito Alves Sousa, Andrey Alves Porto, Yasmim Mota de Moraes Pontes, Simone Sakagute Tavares, Guilherme da Silva Rodrigues, Leonardo Santos Lopes da Silva, Leonardo da Silva Goncalves, Carolina Scoqui Guimaraes, Macário Arosti Rebelo, Andressa Crystine da Silva Sobrinho, Jose E. Tanus-Santos, Vitor Engracia Valenti, Bruno Gualano, Carlos Roberto Bueno Júnior

**Affiliations:** 1 Department of Internal Medicine, Ribeirão Preto Medical School, University of São Paulo, Ribeirão Preto, SP Brazil; 2 Ribeirao Preto Nursing School, University of São Paulo, Ribeirão Preto, SP Brazil; 3 Movement Sciences, Autonomic Nervous System Center (CESNA), São Paulo State University (UNESP), Presidente Prudente, SP Brazil; 4 Ribeirao Preto School of Physical Education and Sports, University of São Paulo, Ribeirão Preto, SP Brazil; 5 Department of Pharmacology, Faculty of Medical Sciences, State University of Campinas, Campinas, SP Brazil; 6 Department of Pharmacology, Ribeirão Preto Medical School, University of São Paulo, Ribeirão Preto, SP Brazil; 7 Autonomic Nervous System Center (CESNA), São Paulo State University (UNESP), Marília, SP Brazil; 8 Applied Physiology & Nutrition Research Group, University of São Paulo, Medical School (FMUSP), São Paulo, SP Brazil

**Keywords:** *Beta vulgaris*, Nitrate, Hypertension, Post-menopause, Cardiovascular physiological phenomena, Sports nutritional sciences

## Abstract

**Background:**

There is no evidence of the use of beetroot juice with a previously recommended dose of nitrate (NO3) (> 300 mg) on the cardiovascular performance during and recovery following exercise in postmenopausal women with systemic arterial hypertension (SAH).

**Methods:**

We will investigate the effects of beetroot juice rich in NO3 acutely (800 mg) and during a week with daily doses (400 mg) on blood pressure, heart rate (HR), cardiac autonomic control, endothelial function, inflammatory, hormonal, and stress biomarkers oxidative stress and enzymes involved in nitric oxide synthesis and mitochondrial regulation, under resting conditions, as well as mediated by submaximal aerobic exercise sessions. Through a randomized, crossover, triple-blind, placebo-controlled clinical trial, 25 physically inactive women with SAH will undergo an acute and 1-week trial, each with two intervention protocols: (1) placebo and (2) beetroot, in which will ingest beet juice with or without NO3 in its composition with a 7-day washout interval. On collection days, exercise will be performed on a treadmill for 40 min at a speed corresponding to 65–70% of VO2peak. The collection of variables (cardiovascular, autonomic, and blood samples for molecular analyses) of the study will take place at rest (135 min after ingestion of the intervention), during exercise (40 min), and in the effort recovery stage (during 60 min) based on previously validated protocols. The collections were arranged so that the measurement of one variable does not interfere with the other and that they have adequate intervals between them.

**Discussion:**

The results of this research may help in the real understanding of the nutritional compounds capable of generating safety to the cardiovascular system during physical exercise, especially for women who are aging and who have cardiovascular limitations (e.g., arterial hypertension) to perform physical exercise. Therefore, our results will be able to help specific nutritional recommendations to optimize cardiovascular health.

**Trial registration:**

ClinicalTrials.gov NCT05384340. Registered on May 20, 2022.

## Administrative information

**Table:** 

Title	Nitrate-rich beet juice intake on cardiovascular performance in response to exercise in hypertensive and postmenopausal women: a randomized, triple-blind, crossover, controlled trial
Trial registration	ClinicalTrials.gov, NCT05384340, registered 20 May 2022.
Protocol version	May 20, 2022. Version 1.
Funding	No funding
Author details	**Cicero Jonas R. Benjamim** University of São Paulo, Brazil, SP, Ribeirão Preto Department of Internal Medicine, Ribeirão Preto Medical School. **Yaritza Brito Alves Sousa** University of São Paulo, Brazil, SP, Ribeirão Preto Ribeirao Preto Nursing School **Andrey Alves Porto** São Paulo State University, UNESP, Presidente Prudente, SP, Brazil. Movement Sciences, São Paulo State **Yasmim Mota de Moraes Pontes** University of São Paulo, Brazil, SP, Ribeirão Preto Ribeirao Preto School of Physical Education and Sports **Simone Sakagute Tavares** University of São Paulo, Brazil, SP, Ribeirão Preto Ribeirao Preto School of Physical Education and Sports **Guilherme da Silva Rodrigues** University of São Paulo, Brazil, SP, Ribeirão Preto Department of Internal Medicine, Ribeirão Preto Medical School. **Leonardo Santos Lopes da Silva** University of São Paulo, Brazil, SP, Ribeirão Preto Ribeirao Preto School of Physical Education and Sports **Leonardo da Silva Goncalves** University of São Paulo, Brazil, SP, Ribeirão Preto Ribeirao Preto School of Physical Education and Sports **Carolina Scoqui Guimaraes** University of São Paulo, Brazil, SP, Ribeirão Preto Ribeirao Preto Nursing School **Macário Arosti Rebelo** State University of Campinas, SP, Campinas **Andressa Crystine da Silva Sobrinho** University of São Paulo, Brazil, SP, Ribeirão Preto Department of Internal Medicine, Ribeirão Preto Medical School. **Jose Eduardo Tanus dos Santos** University of São Paulo, Brazil, SP, Ribeirão Preto Department of Pharmacology, Ribeirão Preto Medical School. **Vitor Engracia Valenti** São Paulo State University, UNESP, Marília, SP, Brazil. Autonomic Nervous System Center, São Paulo State **Bruno Gualano** University of São Paulo, Medical School (FMUSP), Brazil, SP, São Paulo. Applied Physiology & Nutrition Research Group. **Carlos Roberto Bueno Júnior** University of São Paulo, Brazil, SP, Ribeirão Preto Ribeirao Preto School of Physical Education and Sports; Department of Internal Medicine, Ribeirão Preto Medical School.
Name and contact information for the trial sponsor	Not applicable, this trial does not have sponsor.
Role of sponsor	Not applicable, this trial does not have sponsor.

## Introduction

### Background and rationale

Physiological changes in the non-reproductive phase of women impose a high risk for the development of systemic arterial hypertension (SAH), given that the abrupt drop in estrogen levels reduces the production of endothelial relaxing factors, such as nitric oxide (NO) [1]. To the detriment of this, the prevalence of SAH in postmenopausal women tends to increase compared to men of the same age. Consequently, females become more prone to cardiovascular and cerebrovascular diseases [2].

Although there has been a great advance in managing the treatment of SAH, the disease control rates in Brazil are still considered unsatisfactory [3]. A recent meta-analysis with national data revealed a variation between 43.7 and 67.5% of SAH control rates in primary care [4]. Therefore, therapeutic adjuvants are necessary for optimizing cardiovascular functioning in SAH, among which beetroot juice has been gaining notoriety.

Betaine and nitrate have better-established purposes among the various nutritional compounds present in beets. Betaine is a potent anti-inflammatory and antioxidant compound [5], in addition to having an essential role in eliminating toxic metabolites from the body (e.g., homocysteine) [6] and reducing the risk of a heart attack myocardial infarction [7].

Nitrate (NO3), when ingested, is partially reduced to nitrite (NO2) by oral bacteria from NO3 reductase. Subsequently, upon reaching the stomach, NO2 reacts with low pH and is transformed into NO, which is easily transported across cell membranes into the bloodstream, where it plays a crucial role in vasodilation. The NO2 remaining in the stomach is absorbed in the initial parts of the duodenum and follows other pathways to increase NO production in the circulation [8]. In addition to the previously described exogenous pathway, NO can also be synthesized endogenously via the l-arginine + O2 pathway, mediated by NO-synthase enzymes [9]. Recent meta-analyses of clinical trials have shown that NO3 consumed through beetroot juice lowers blood pressure more clearly [10, 11] than in its salt form (e.g., sodium/potassium NO3) [12]. There are assumptions that this occurs through the interaction between NO3 and the vitamins, minerals, and phenolic compounds present in beet juice, generating more intense pressure and vascular responses [10].

Considering that the endogenous production of NO is reduced with aging [13], it was recently confirmed that the hypotensive effects caused by beetroot juice are more evident in older adults (50 to 70 years of age) when compared to a young population (18 to 30 years of age) [14]. In addition, a meta-analysis recently published by our group showed that in patients with SAH, NO3-rich beetroot juice supplementation was able to reduce systolic blood pressure by − 4.95 mmHg (95% CI: − 8.88; − 1.01) without significant differences to diastolic blood pressure [10].

Accordingly, physical exercise is considered one of the non-drug therapeutic strategies that directly helps to reduce blood pressure levels by causing post-exercise hypotension (PEH) [15] and contributing to increased baroreflex sensitivity [16]. HPE has excellent clinical value for hypertensive patients, as it can be used as a strategy to reduce blood pressure levels both acutely and chronically. Aerobic exercise is considered one of the most efficient to generate PEH [17–19].

In conjunction with heart rate (HR) and blood pressure (BP) levels, heart rate variability (HRV) has been used as a non-invasive and independent metric for assessing cardiovascular risk [11, 20, 21]. Through the adjacent intervals between consecutive heartbeats (RRi), it is possible to analyze the behavior of autonomic efferents (parasympathetic and sympathetic) on the heart [22, 23]. Part of the effects caused by hypertension on cardiac activity involves adjustments in the autonomic nervous system (ANS), and therefore, in hypertensive patients, there is a decrease in HRV at rest [24], and after exercise, there is a delay in recovery of parasympathetic modulation [25]. These two factors are evidence of autonomic dysfunction and cardiovascular inadaptability that certainly contribute to the emergence of disorders and adverse cardiovascular events [26] and a risk factor for overall mortality [27]. In SAH, endothelial dysfunction is documented in the macro- and microcirculation of the coronary, peripheral, and renal circulation. These effects are mainly caused by decreased endogenous nitric oxide production through increased oxidative stress and endothelial damage caused by increased blood shear force [28].

To date, studies that have investigated the effects of NO3-rich beet juice on the cardiovascular exercise responses of hypertensive patients applied it in an acute dosage of 35 mL and, therefore, with a limited amount of NO3 (200 mg) [29, 30]. But these dosages are at odds with the pharmacodynamic and dose-response studies of beet juice rich in nitrate, and this may be the main factor why there are no summative effects on the hypotensive response [29] and autonomic recovery [30] after exercise were found in women with arterial hypertension. It has already been shown that only after 4 to 6 days of beet juice ingestion (> 300 mg of nitrate) the bioavailability of inorganic NO3 is increased to cause physiological effects [31]. Furthermore, the study by Wylie et al. [32] determines that an optimal acute dosage of beetroot juice for cardiovascular outcomes is 140 mL, that is, approximately 800 mg of nitrate. Our study aims to resolve these issues by applying the dosages recommended by the studies by Wylie et al. [32] and Bailey et al. [31].

### Objectives and hypotheses

The objective is to investigate the effects of beetroot juice rich in NO3 acutely (800 mg) and for a week with daily doses (400 mg) on blood pressure; heart rate; cardiac autonomic control; endothelial function; inflammatory, hormonal, and oxidative stress biomarkers; and enzymes involved in nitric oxide synthesis and mitochondrial regulation, under resting conditions, as well as mediated by submaximal aerobic exercise sessions.

We hypothesize that interventions with NO3 from the BRJ, both acutely and during a 1-week intervention, will improve cardiovascular parameters in response to exercise and in the recovery from the submaximal exercise test.

### Trial design

The study is classified as a randomized, crossover, triple-blind, placebo-controlled clinical trial and will have two arms. The study protocol is registered on the ClinicalTrials.gov platform (NCT05384340) and described according to the Standard Protocol Items: Recommendations for Interventional Trials (SPIRIT) checklist. The results from this clinical trial will be described following the Consolidated Standards of Reporting Trials (CONSORT) guidelines.

## Methods: participants, interventions, and outcomes

### Study setting

The study will be conducted at the Laboratory of Exercise Physiology and Metabolism (LAFEM) of the School of Physical Education and Sport of Ribeirão Preto, University of São Paulo (EEFERP-USP).

Participants will visit the laboratory seven times for the familiarization, collection, and measurement of variables. Sessions will be held between 7:30 am and 11:00 am to standardize the influence of the circadian rhythm on the parameters evaluated. The environment will be controlled with temperature between 22 and 25 °C and humidity between 60 and 70%.

On collection days, 2 h before data measurement, participants already in the laboratory will ingest beetroot juice (Beet It, James White Drinks, Ltd.) with or without nitrate in its composition. The juices will be identical in their organoleptic characteristics, and therefore, only the amount of nitrate will differ between them.

### Eligibility criteria

Women aged between 50 and 70 years living in the city of Ribeirão Preto, São Paulo, Brazil, with a previous diagnosis of SAH [3], will be recruited. Study participants must be physically inactive according to the Modified Baecke Questionnaire for the elderly (QBMI) [33]. We will consider the systolic blood pressure values between 120 and 159 mmHg or diastolic blood pressure values between 80 and 99 mmHg [34]. To be eligible to participate in the study, participants must have a diagnosis of post-menopause (amenorrhea for a period equal to or greater than 12 months) [35], have health conditions to practice physical activity according to the Physical Activity Readiness Questionnaire (PAR-Q), and not having a history of previous acute myocardial infarction or stroke confirmed by medical history, in addition to having no known allergy and/or intolerance(s) to nitrate, milk, and gluten. Those that interfere with the pH of the stomach (e.g., proton pump inhibitors) and/or using drugs that directly affect cardiac autonomic control (e.g., beta-blockers and calcium channel antagonists) will be excluded from the study. The other medications used by the participants will be recorded to be included as adjustment variables in the statistical analysis.

### Interventions

#### Intervention description

The purpose of the study is to identify the effects of NO3 from beetroot juice, and therefore, the study was divided into two arms: (a) beetroot juice rich in nitrate and (b) beet juice without nitrate. The other protocols will be applied to the participants at the exact times and times of the day. In addition, because it is a crossover study, all participants will undergo all interventions equally.

Each protocol will last for 7 days, and its order of execution will be established through a randomization process. As this is a crossover study, each participant will participate in both protocols. Between one protocol and another, a 7-day washout interval will be given for the purification of the compounds provided by the protocols. Subsequently, the intervention contrary to the first will be provided, with the dynamics of the intervention being the same for both phases. Participants will receive eight units of juice bottles corresponding to the first intervention (placebo or nitrate) on the first laboratory visit. On the first day of intervention of each protocol, they will take two units (140 mL–800 mg of NO3), and from the second to the seventh day, they will take one unit (70 mL–400 mg of NO3) per day. Of the eight units (100%), six bottles (75%) will be destined for the participants to take home. On the seventh day of the intervention, the participants will take the last unit of juice at home and return to the laboratory for the last evaluation.

On the days that they do not visit the laboratory and on the last day of evaluation, the participants will drink the juice in the morning and on an empty stomach before brushing their teeth. After drinking the juice, we will ask them to take a 30-min break to consume breakfast. Photographs or videos of the participants ingesting the juice will be requested daily and sent to the researcher.

Participants will not be informed about the order of administration of interventions. The researcher responsible for collecting and measuring the variables studied will also be blinded so as not to know which protocol will be administered. An independent researcher will be responsible for taking the juice and delivering it to the responsible researcher. In addition, another independent researcher/collaborator will be responsible for the statistical analysis of the data, so that he will be blinded so that he is not aware of which protocol order will be analyzed. Based on the steps previously described, the design of a randomized, triple-blind, placebo-controlled trial will be achieved. The quality of blinding will be assessed at the end of the study by asking each participant which beverage they believe they are ingesting in each of the two protocols.

#### Criteria for discontinuing or modifying allocated interventions

No criteria will be determined for discontinuing or modifying the allocation order. Participants who do not comply with the interventions will also be evaluated and will provide information about when they abandoned the intervention for later accounting. All of them will have their variables analyzed by intention to treat.

#### Strategies to improve adherence to interventions

A trained researcher will be responsible for supporting participants who may find it challenging to adhere to the study interventions. Before obtaining consent from participants to participate in the research, we will provide information about the research and its relevance to their health during the experimental procedures. In addition, the research team will be trained to be available to resolve doubts. At the end of the study, the participants will be invited to participate in a physical training and nutrition group for the elderly at the Escola de Educação Física e Esporte de Ribeirão Preto, USP, Brazil.

#### Relevant concomitant care is permitted or prohibited during the trial

Prior to carrying out the stages of the experimental procedures, the participants will receive instructions and guidelines to fill in a food diary on the days prior to measuring the variables of each intervention protocol, the foods, and amounts ingested from breakfast (first meal) until supper (last meal). We will ask the participants to eat the same foods and in similar amounts on the day before each collection. According to the study by Griesenbeck et al. [36], a list of foods that are considered high in nitrate and participants will be instructed not to ingest during the study. The amount of carbohydrate, protein, fat, and nitrate intake will be assessed by a trained nutritionist through a specific software (Nutritionist Pro® v. 7.3, Axxya Systems, Woodinville, WO, USA). Potential drug-food interactions are possible, but it is difficult to tease these out in our study; however, participants’ food consumption and drug regimen will be well characterized, and our cross-over design will minimize the impact of such potential interactions on intraindividual responses, as the each participant will undergo all different conditions (placebo and experimental). On the days of the laboratory visit, not having breakfast as a standard meal will be provided 40 g of whey protein (Top Whey 3w, Max Titanium®, SP, Brazil) before executing the experimental protocols. Avoid performing vigorous physical efforts the day before and on each experiment; we will ask participants not to change your physical activity habits while participating in the study. Do not drink caffeine-based beverages and other stimulants or alcoholic beverages within 12 h and 24 h, respectively, prior to each stage of the study, and do not use mouthwash during all experimental protocols and between them in order to avoid the reduction of the oral microbiota responsible for nitrate metabolism [37].

#### Initial evaluations

Participants will be identified by collecting the following information: age (years), body mass (kg), height (cm), body mass index (BMI), hip circumference (HC), waist circumference (WC), and circumference calf (CP). Anthropometric measurements will be obtained according to the recommendations described by Lohman et al. [38]. BMI will be calculated using the following formula: body mass (kg)/height^2^ (m). The waist/height ratio will be calculated by dividing the waist circumference by the height obtained in centimeters. The following assessments will also be carried out:

1. Body composition: The fat percentage of the participants will be collected for a better characterization of the studied sample. The dual X-ray absorptiometry (DXA) will analyze the body composition, as lean body mass and fat mass. The exam will be conducted by a clinician trained in radiology at the Laboratory of Cineantropometry and Human Development [39].
2. Familiarization of participants for the VO2peak test: A familiarization protocol will be used to improve the reliability of the data obtained with the VO2peak test. The participants will be physically inactive and will not be acclimated to the physical exercise, which may underestimate the values obtained in the test. At this stage, as a way of monitoring and safety of the participants, three collections of systolic and diastolic blood pressure will be performed before any procedure. On the first day, we will collect blood (15 mL) to analyze the blood glucose and lipid profile. We will use these data to characterize the sample. Prior to the VO2peak determination test, the participants will undergo a familiarization phase with the treadmill during three sessions performed on three different days. The Bruce protocol will be applied to determine the VO2peak. The values obtained during this phase will not be considered in the study [40].
3. Determination of VO2peak (maximum exertion test): Another day will be scheduled to perform the VO2peak test of the participants so that the participants come to the laboratory in the fed state and with light clothes. The test will be performed on a treadmill (TPEE; Inbrasport ATL 2000®) using the Bruce protocol for the consequent use of 65–70% of the speed corresponding to the VO2peak for the load applied in the following steps. For VO2peak analysis, expired gases are analyzed in a commercial Teem 100 - VO 2000 system (Aerosport, Ann Arbor®, USA) previously validated by Novitsky et al. [41] and Wideman et al. [42] and periodically calibrated with volumes and gases of known concentration.

#### Exercise variables

**Fig. 1:**
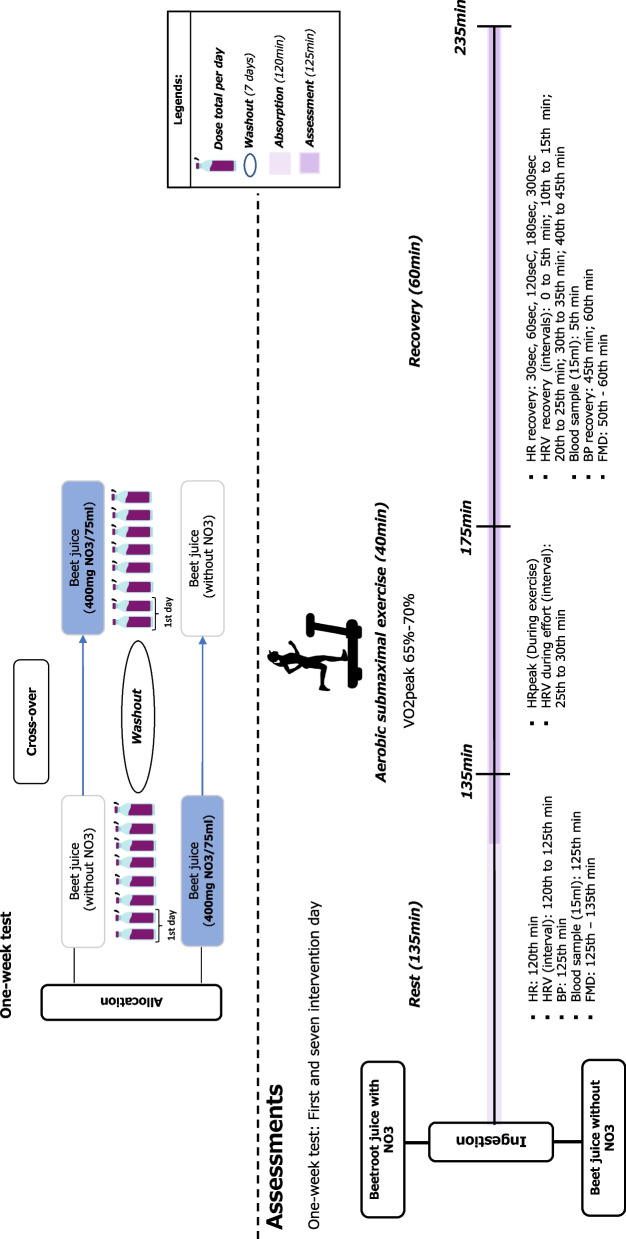
Experimental procedures of the study and collecting data

On the first day, the collections will occur after the participants ingested 140 mL of beet juice (with or without nitrate). The seventh day of beet juice ingestion (70 mL) (with or without nitrate) will also be collected. On the collection days (first and seventh days of intervention), the variables will be obtained at the following times (Fig. 1):

I) Rest phase (zero to 135th min): After drinking the juice, the participants will remain at an initial rest in a sitting position. Then, 30 min before starting the measurements, the participants will be instructed to empty their bladders. Upon returning to the room, a recording strap (Polar® H10) will be placed on the chest, in the region of the distal third of the sternum, to record beat-to-beat HR throughout the collection, mediated by a heart rate monitor (Polar® RS800CX, Finland). Then, the participants will be placed on a stretcher in the supine position and will remain at rest. After the remaining time, HR values will be recorded (120th minute), HRV recording (120th to 125th min), systolic blood pressure (SBP) (125th minute), diastolic blood pressure (DBP) (125th minute), blood collection (15mL) (125th min), and analysis of endothelial function (flow-mediated dilation) (125th to 135th min).
II) Physical exercise (135th to 175th min): Participants will perform the physical exercise for 40 min on a treadmill at a speed corresponding to 65–70% of VO2peak [29]. In this phase, the highest HR value reached during the exercise will be recorded, and the HRV recording will also be carried out in the last 5 min (35th–40th min).
III) Recovery phase (175th to 235th min): At the end of the activity, the participants will be placed in the supine position again and will be monitored for another 60 min, recording their HR at 30 s, 60 s, 120 s, 180 s, and 300 s. Blood collection (15 mL) will be performed between 5 and 10 min after exercise. HRV will be recorded after exercise at the following times: 0–5 min, 10–15 min, 20–25 min, 30–35 min, and 40–45 min. SBP and DBP will be recorded after exercise at the 45th minute and 60th minute post-exercise. To avoid errors in the measurements of the evaluated parameters, a single evaluator will perform these measurements throughout the experiment. The flow-mediated dilation technique to assess endothelial function will be employed after exercise during the 50th–60th minute post-exercise interval.

### Primary outcomes

#### Blood pressure

The measurement of systolic and diastolic blood pressure will be performed indirectly using a digital sphygmomanometer (OMRON M2, Model HEM-7121-E, Jundiaí, São Paulo, Brazil) previously calibrated on the participants’ left arm.

### Secondary outcomes

#### Heart rate variability analysis

For the analysis of HRV indices, heart rate will be recorded beat by beat throughout the experimental protocol with a sampling rate of 1000 Hz. Stable series with sections of 256 RR intervals will be selected. The last 256 R-R intervals of each 5-min recording window will be analyzed [43]. In these series, digital and manual filtering will be performed to eliminate premature ectopic beats and artifacts. Only those with more than 95% of sinus beats will be included [44].

#### Linear indices

The time-domain analysis will be performed using the standard deviation of the mean of normal R-R intervals (SDNN) and square root of the mean of the square of differences between adjacent normal R-R intervals (RMSSD) indices. For the analysis of linear indices in frequency and time domains, the program Kubios HRV 2.1 analysis® will be used [45]. High-frequency spectral components (HF: 0.15–0.40 Hz) in absolute units (ms^2^) will be used to analyze HRV in the frequency domain. We will choose not to evaluate the low-frequency index (LF: 0.04–0.15 Hz) and the LF/HF ratio since the scientific literature demonstrates that its concept is defective and lacks empirical support to characterize vagal balance—sympathetic [46]. The spectral analysis will be calculated using the fast Fourier transform.

#### Ultra-short-term analysis

Ultra-short-term analyses will be performed with the RMSSD index. For a more detailed analysis of the reactivation of parasympathetic flow after exercise, the last 60 intervals of the resting recording will be selected for comparison with the first 5 min post-exercise divided into 60 cardiac intervals.

#### Heart rate recovery analysis

Heart rate (HR) will be recorded continuously at rest (HR rest), during exercise, and recovery. Resting HR will be measured immediately before the test, with the participants still standing on the treadmill. Reserve HR will be calculated as the difference between peak HR during exercise and at rest. The chronotropic response (CR%) will be calculated using the following formula: ([HRpeak − HRrest/220 – age − HRrest] × 100). HR recovery (HRrec) will be calculated as the difference in HR after the 30 s, 60 s, 120 s, and 180 s of recovery concerning HRpeak during exercise (HRrec30s, HRrec60s, HRrec120s, and HRrec180s, respectively). A reduced HRrec after exercise will be established when differences ≤ 12 bpm are found in HRrec60s [27].

#### Endothelial function

The flow-mediated dilatation (FWD) technique will be performed with a bidirectional ultrasound device (2D), with color and spectral Doppler and linear probe transducer with a frequency of 14 Mhz from the Toshiba® brand. The technique of Celemajer et al. [47]. First, the participant will be positioned in dorsal decubitus (supine position) with controlled abduction of the right arm. The linear transducer will be positioned in the medial phase of the arm to obtain a longitudinal image of the right brachial artery in B-mode (parallel to the transducer), 5–10 cm to superior the antecubital crease.

After measuring the basal diameter (D1), the transducer location will be marked with an anthropometric evaluation marker (Penta Texta 700 Fine) so that the post-occlusion diameter measurement can be performed at the same location. Subsequently, the brachial artery will be occluded for 5 min, with a pressure cuff placed on the arm with a pressure adjustment slightly higher than the SBP. After 60 s of cessation of inflation, the artery’s post-occlusion diameter (D2) will be measured. The measurements of D1 and D2 will be carried out in the same place in the vessel. The DILA will be calculated by the formula (DILA (%) = D2 − D1/D1 × 100).

#### Blood tests

##### Quantification of plasma concentrations of nitrite

Plasma concentrations of nitrite will be used to indirectly quantify the changes in nitric oxide (NO) concentration. This analysis will be necessary before and after exercise to attest that the possible effects found will be due to increased nitrate intake, which was later converted into nitrite. The Nitric Oxide Analyzer Sievers device (NOATM) – model 280i, USA, will be used. In the sealed glass chamber of the device, 300 μL of blood samples will be injected in contact with 8 mL of acidified triiodide solution (2 g of potassium iodide and 1.3 g of iodine dissolved in 40 mL of water with 140 mL of acid acetic). NO will be produced in the form of gas by the reaction of nitrite with the iodide solution. A continuous flow of nitrogen gas carries NO to the other chamber for reaction with ozone—the detection of NO in the device is based on the gas-phase reaction of NO with ozone [48].

##### Quantification of plasma lipid peroxidation by MDA

Oxidative stress will be estimated through the secondary products of lipid peroxidation and will be analyzed through the quantification of Malondialdehyde (MDA). MDA is considered a reliable general biomarker of plasma oxidative damage. The quantification of MDA will be performed using high-performance liquid chromatography (HPLC), with visible detection (VIS), according to the methodology of Grotto et al. [49]. After the centrifugation step, the plasma will be separated. In the volume of 75 μL of plasma, 25 μL of water and 25μL of NaOH3N will be added, which will be incubated for 30 min in a horizontal agitation system with heating at 60 °C. Afterward, 125 μL of 6% H3PO4 and 125 μL of 0.8% TBA will be added to the samples, and the product will be incubated for 45 min at 90 °C. Then, 50 μL of 10% sodium dodecyl sulfate (SDS) will be added to the samples. The samples will be extracted with 300 μL of n-butanol after 1 min of vortexing and will be centrifuged at 3000*g* for 10 min; 20 μL aliquots of the organic phase will be injected into the chromatograph [49].

##### Quantification of plasma non-enzymatic antioxidant defenses by FRAP

This quantification will be done by the ferric reducing ability of plasma (FRAP) technique, whose principle is based on reducing the ferric ion Fe3+ to ferrous Fe2+. For this, three solutions will be prepared: A (acetate buffer: 300 mM, pH 3.6, and 40 mM HCl), B (TPTZ (2,4,6-tri[2-pyridyl]-s-triazine), 10 Mm), and C (ferric chloride hexahydrate (FeCl3.6H2O), 20 mM), forming the working reagent A + B + C in the proportion 10:1:1 (V/V). Then, 80 μL of the plasma obtained will be added to a mixture of deionized water (250 μL) with the working reagent (2.4 mL). This solution containing the sample will be incubated in a water bath at 37 °C for 15 min in the dark. Then, the FRAP concentrations will be estimated by interpolating the absorbances determined in the samples with those determined in a standard curve, which will be prepared by diluting a standard solution of ferrous sulfate (Fe11) in distilled water, obtaining final concentrations of 0, 31.25, 62.5, 125, 250, 500, and 1000 μmol/L. Afterward, this solution will be placed in a microplate. The absorbances (at 593 nm) will be read in parallel with a standard curve of ferrous sulfate using a microplate reader spectrophotometer [50].

##### Hormones, catecholamines, inflammatory, and mitochondrial markers

We will evaluate the concentrations of molecules directly and indirectly associated with the cardiovascular system before and after using beet juice rich in nitrate. These analyses allowed us to verify the influences and the relationships that inflammation markers, hormones, and catecholamines have with the cardiovascular system on the effects of beet juice rich in nitrate. Plasma concentrations of leptin, ghrelin, adiponectin, lipoprotein lipase, and hormone-sensitive lipase will be determined from a quantitative enzymatic assay using specific commercial kits (Linco Research Inc., St. Louis, MO, USA). The analysis will be carried out to evaluate concentrations of the interleukins (IL-6 and IL-10), tumor necrosis factor-alpha (TNF-α), interferon-y (IFN-y), C-reactive protein (CRP), nuclear factor-kappa B (NF-kB), vascular endothelial growth factor (VEGF), monocyte chemotactic protein 1 (MCP-I), vascular cell adhesion molecule 1 (VCAM-I), intercellular cell adhesion molecule-1 (ICAM-I), and N-terminal portion of the pro-hormone natriuretic peptide (NT-proBNP) (Biolegend, San Diego, CA, USA). Plasma catecholamines (epinephrine and norepinephrine) will be tested using a commercially available dual-purpose enzyme-linked immunosorbent assay (ELISA) kit (ABNOVA, Taiwan) [51]. Mitochondrial biogenesis will be evaluated by quantifying factor-1 and factor-2 markers related to NF-E2 (NRF1 and NRF2) by peroxisome proliferator-activated receptor gamma coactivator 1-alpha (PGC-1α), using an ELISA kit specific (ABCAM, USA) [52].

### Participant timeline

**Fig. 2:**
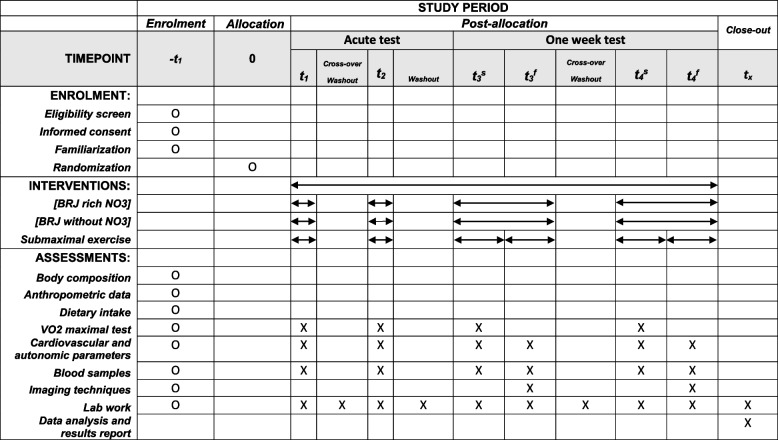
Recommended SPIRIT figure with the participant timeline

Figure 2 shows the recommended SPIRIT figure with the participant timeline.

### Sample size

The number of participants in this study considered the most recent meta-analysis study, which found a reduction of 5 mmHg caused by beetroot juice rich in nitrate in patients with SAH as significant with a variation of 4 mmHg [10, 11]. We applied these values in the Hedwig Harvard Sample Calculator software online, considering a bilateral 95% confidence interval and a power analysis of 80% that provided a minimum of 11 participants for this clinical trial [29]. We will recruit sixteen postmenopausal women diagnosed with SAH to ensure a sufficient sample size after the interventions. This sample size is consistent with previous studies that investigated the effects of BRJ on BP in patients with SAH. The Amaral et al. [29] study used 13 postmenopausal women to examine the impact of BRJ on PEH. Broxterman et al. conducted two studies in patients with SAH medicated and non-medicated. In both trials, the sample was 13 and 14, respectively. The most recent investigation in this field conducted by Siervo et al. [53] used 16 patients with SAH.

### Recruitment

Recruitment will take place on a monthly basis. The dissemination of the study and recruitment will take place through media and communication channels. We will use local radio stations to publicize the study. In addition, we will post on social media (e.g., Facebook, Instagram) on research group pages and researchers’ personal profiles.

### Assignment of interventions: allocation

#### Sequence generation and implementation

**Fig. 3:**
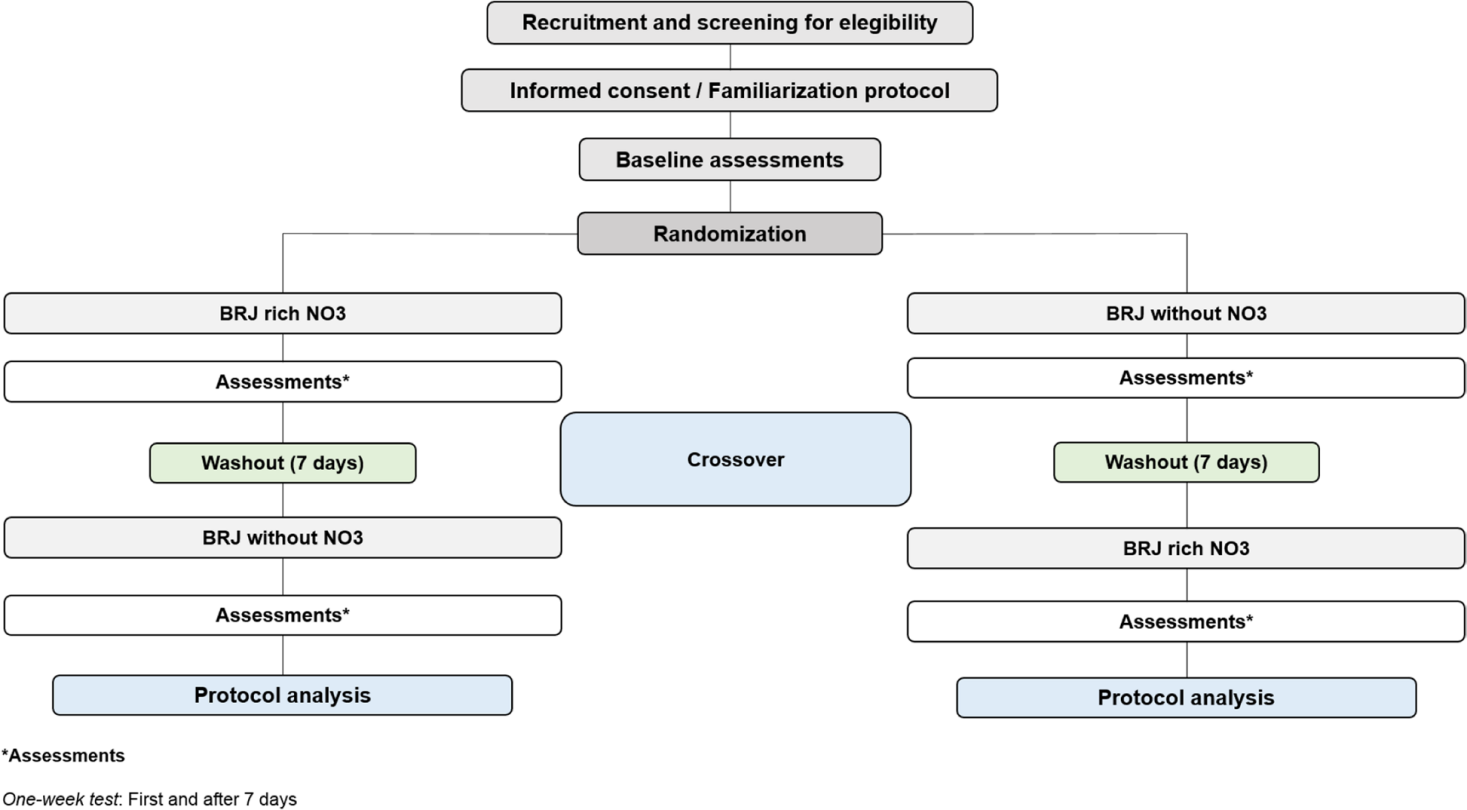
Study design flowchart

We will use the randomizer.org website to randomize the order of intervention that participants will receive. For each treatment, 16 “sets” (total number of participants) will be created with two treatment options, “number 1” or “number 2.” Each number refers to a type of protocol and, therefore, will be previously coded by a researcher who will not participate in the experiments and the treatment allocation order will be respected (e.g., set 1—number 1; set 2—number 1; set 3—number 2; [...] set 16—number 1). After the end of the first intervention, the participants will perform the opposite intervention (Fig. 3).

### Assignment of interventions: blinding

#### Who will be blinded

As this is a triple-blind study, clinical trial participants, researchers, and the outcome assessor will be blinded. Interventions will be codified by random letters or numbers, and a researcher not responsible for data collection will be responsible for keeping this complete information confidential. Only after data collection, processing, and analysis will the interventions be revealed. We do not anticipate any requirement for unblinding, but if required, the researchers will have access to group allocations and any unblinding will be reported.

### Data collection and management

#### Plans for assessment and collection of outcomes

Interventions and evaluations will be carried out at the Laboratory of Exercise Physiology and Metabolism at EEFERP. All assessments will be conducted by at least a physical educator, nutritionist, and nurse, trained and familiarized with the assessment protocol prior to the study.

#### Plans to promote participant retention and complete follow-up

The researchers will be in daily contact with the study participants. Your health status will be routinely asked, and survey requirements will be reinforced. Forty-eight and 24 h before assessments, text messages will be sent to remind participants of the assessment date and time. If any participant misses the assessment, we will immediately contact a call to inquire about the reasons for the no-show.

#### Data management

The information collected in this clinical trial will always be protected and in the care of the principal investigator. The data will be kept in your office and all investigation documents to ensure data security. Research information, when stored electronically, will have a copy stored on a drive that will be retained with a password for access and under the protection of the principal investigator (CRBJ).

### Statistical methods

For data analysis, descriptive statistics will be performed to characterize the sample. The results will be presented with mean and standard deviation values when the residual values of the variables present normal. Values in the median distribution and interquartile intervals will be presented when the residuals’ assumptions of normality and homoscedasticity are not reached. Data will be analyzed using linear analysis of mixed effects adjusted for age, drugs, and VO2peak, considering time and treatment as an independent variable. Per-protocol analysis will be performed to account for possible dropouts during the study. The analysis will be conducted according to the intention-to-treat principle. The generalized mixed linear model will treat the missing values, using maximum likelihood to estimate the model parameters. Differences in all tests will be considered significant when the *p* value is less than 0.05. The statistical program used will be RStudio (v. 1.0.136). The confidence interval at the 95% level will be exposed when we describe the comparison values of the variables with each other. The magnitude of significant differences will be quantified through effect size calculated using Cohen’s *d*. A large effect size will be considered for values greater than 0.9 and a medium effect size for values between 0.9 and 0.5 [54].

### Oversight and monitoring

#### Composition of the coordinating center and trial steering committee

The lead researcher will coordinate the study site. The study will be led by principal investigators. No additional steering committees are considered for this study. The other researchers will participate in regular (monthly) and extraordinary meetings (when necessary) to discuss the progress of the research and possible unforeseen events. We will have two specific researchers available all the time to ensure the participants’ security and that all adverse events, symptoms, and feelings will be reported in this clinical trial. This Trial Committee will be available to participants day-to-day report your symptoms. Furthermore, they will provide further health assistance and guidance to participants searching the health system if necessary.

#### Composition of the data monitoring committee, its role, and reporting structure

The lead researcher will coordinate the study site. The study will be led by principal investigators. No additional steering committees are considered for this study. The other researchers will participate in regular (monthly) and extraordinary meetings (when necessary) to discuss the progress of the research and possible unforeseen events. We will have two specific researchers available in person or by telephone to ensure the participants’ safety and that any potential adverse events and symptoms will be properly collected. Furthermore, these researchers will provide immediate guidance to the participants in need of health assistance for any reasons associated with their participation in the study.

#### Adverse event reporting and harms

Although the interventions used have a minimal risk of causing adverse events, during experimental procedures, participants will often be asked about any change in their well-being or in their perception of their health status. Any symptoms and adverse effects will be reported in the final publication of the manuscript.

#### Frequency and plans for auditing trial conduct

The principal investigators will continuously monitor the conduct of the study. No further monitoring will be carried out unless requested by the USP School of Physical Education and Sports Research Ethics Committee.

#### Plans for communicating important protocol amendments to relevant parties (e.g., trial participants, ethical committees)

If, by chance, there are changes in the research protocol, these changes will be informed and analyzed by the Research Ethics Committee of the School of Physical Education and Sports at USP, which is responsible for the evaluation and approval of the experimental procedures. If the institutional ethics committee team approves, the modifications will be inserted into the study and updated on the clinicaltrials.gov platform by the CJRB principal investigator.

#### Who will take informed consent?

Informed consent will be obtained through the participants’ signature, freely agreeing with their participation in the study. The free and informed consent form was approved by an Ethics and Research Committee (attached). This document contains all the rights of the participants, including withdrawing at any time without loss to themselves.

#### Additional consent provisions for the collection and use of participant data and biological specimens

Upon submission to the ethics committee, the creation of the “BEETMHP5070” biorepository was requested to store biological samples (e.g., blood) that will be collected during the study. In the consent form, participants may or may not allow these biological samples to be used for further analysis in the future.

#### Confidentiality

To maintain confidentiality during and after the assessments, an independent researcher will store participant data separately from any identifying information. Data will be encrypted with a unique identity in a password-protected local database. Only that researcher and the study supervisor will have access to the information on the device.

#### Provisions for post-trial care

Participants will be invited to participate in a nutrition and physical education and exercise program for the elderly, in which they will receive instructions to perform physical activity accompanied by a physical educator and will also receive instructions and guidelines for healthy eating.

#### Dissemination plans

Each participant will receive a complete report with the results of their health parameters after the investigations. At the end of the clinical trial, the research team will contact the participants to provide the study’s final results and deliver educational material with information on healthy eating and exercise recommendations. The study results will be written and analyzed to be published in an indexed scientific journal with a recognized editorial board and peer review within five years after the last participant’s enrollment. Preliminary and secondary results will be presented at local and international conferences.

#### Plans to give access to the full protocol, participant-level data, and statistical code

Not applicable. Public access to the complete protocol, datasets, and statistical code is not planned for this study. However, this information may be available upon reasonable request to the corresponding author, maintaining the participant’s anonymity.

#### Plans for collection, laboratory evaluation, and storage of biological specimens for genetic or molecular analysis in this trial/future use

The biological material that will be collected during the experiments will be blood samples from the participants. A part of the sample content will be used to analyze molecules directly and indirectly associated with the cardiovascular system. Further analyses in the future may be carried out with these samples. However, prior to this, a new approval of the ethics committee will be necessary with the new analyzes that will be analyzed. Only those participants who have agreed to have their samples used in the future will be included in the analyses. The other samples will be discarded at the end of the experiments listed in this protocol. The research ethics committee approved the creation of the BEETMHP5070 Biorepository intended to store eligible blood samples.

#### Patient Public Involvement

There was no public or patient involvement in the design of this protocol.

## Discussion

Our study intends to shed light on the evidence of the effects of beet juice rich in NO3 at rest and after cardiovascular stress caused by submaximal aerobic exercise. The choice to test this intervention in postmenopausal women with SAH is due to this population’s high cardiovascular risk.

The strength of this clinical trial includes the randomized and controlled protocol, which will make it possible to identify the real effects of this nutritional intervention. In addition, a triple-blind model was incorporated to ensure that the results achieved have better reliability. The duration of the intervention chosen is following classical studies that have determined the best dosage for the effects of beetroot juice rich in NO3 to begin to appear, both acutely (140 mL [800 mg NO3] of juice) and continuously (4–6 days of intervention × 70 mL [400 mg] of juice) [31, 32].

A previous study investigating the acute effects of BRJ rich in NO3 did not find an extension of the submaximal aerobic exercise in post-exercise hypotension [29]. Furthermore, the HRV recovery shows no difference compared to the placebo group [30]. According to previous studies in this field [31, 32], an acute optimal dose of BRJ rich in NO3 to generate cardiovascular effects in exercise appears to be around 140 mL or 800 mg NO3. In Amaral et al. [29], the authors reported using a dosage of 400 mg, but they provided only 35 mL of BRJ (equal to 200 mg). These NO3 concentrations seem low to an acute test and may explain the absence of effects. Despite that, the Amaral et al. [29] study is a well-conducted and controlled trial demonstrating that NO3 dosage between 200 and 400 mg can be inefficient in extending the drop BP after aerobic exercise or promoting quick recovery of HRV [30].

Our study protocol brings novelties, due to the analyses that will be employed in the study being carefully chosen to generate an understanding of the effects of ingesting the juice rich in NO3 at optimal dosages. This research may help in the real understanding of nutritional compounds capable of generating safety to the cardiovascular system during physical exercise, especially for women who are aging and who have cardiovascular limitations (e.g., arterial hypertension) to perform physical exercise. Our results will be able to build nutritional recommendations based on beetroot juice to optimize heart health.

The article presents the schedule of cardiovascular variables after physical exercise that may be useful for other studies. We strive to create a research protocol with a vast collection of variables. Furthermore, carefully, the sequence of collecting variables will not interfere with the results of the other. This study protocol provides a useful design to improve the collection data in studies focusing on assessing cardiovascular stress post-exercise, such as heart rate variability, post-exercise hypotension, heart rate recovery, and flow-mediated dilation.

### Trial status

Recruiting.

Version 1. May 20, 2022.

Date recruitment began: May 20, 2022.

Approximate date when recruitment will be completed: January 31, 2023.

## Data Availability

After the study publication, the data and materials will be available upon a reasonable request to the corresponding author.

## Abbreviations

HRV: Heart rate variability
HR: Heart rate
BP: Blood pressure
SBP: Systolic blood pressure
DBP: Diastolic blood pressure
ANS: Autonomic nervous system
BRJ: Beetroot juice
NO2: Nitritre
NO3: Nitrate
NO: Nitric oxide
Interleukin 6: IL-6
Interleukin 10: IL-10
TNF-a: Tumor necrosis factor-alpha
VEGF: Vascular endothelial growth factor
IFN-y: Interferon-y
MCP-I: Monocyte chemotactic protein 1
VCAM-I: Vascular cell adhesion molecule 1
ICAM-I: Intercellular cell-adhesion molecule-1
NF-kB: Nuclear factor-kappa B
CRP: C-reactive protein
NT-proBNP: N-terminal portion of the B-type natriuretic peptide prohormone
GLP-1: Glucagon-like peptide 1
eNOS: Endothelial nitric oxide synthase
PGC-1α: Peroxisome proliferator-activated gamma receptor coactivator 1-alpha
PEH: Post-exercise hypotension
NRF1: NF-E2 related factor-1
NRF2: NF-E2 related factor-2
RMSSD: Root mean squared standard deviation of R-R intervals
LAFEM: Lab exercise physiology and metabolism
EEFERP: Ribeirão Preto Physical Education and Sports School
QBMI: Baecke Questionnaire Modified for Elderly
SBC: Brazilian Cardiology Society
SAH: Systemic arterial hypertension
ACMS: American College of Sports Medicine
USP: University of São Paulo
FMRP: Ribeirão Preto Medical School
TCLE: Informed consent term
